# Anti-Inflammatory Properties of Curcumin as Add-On Therapy in Patients with MS—Prospective, Comparative, Randomized, Pilot Study

**DOI:** 10.3390/pharmaceutics18050519

**Published:** 2026-04-24

**Authors:** Anna Kukushkina, Vladimir Rogovskii, Olga Zhilenkova, Timur Sadekov, Mikhail Melnikov, Alexey Boyko

**Affiliations:** 1 Department of Neuroimmunology, Institute of Clinical Neurology, Federal Center of Brain Research and Neurotechnologies of the Federal Medical Biological Agency, 117513 Moscow, Russia; dr_kukushanna@mail.ru (A.K.);; 2 Zhadkevich Moscow City Clinical Hospital, 121374 Moscow, Russia; 3 Department of molecular pharmacology, N.I. Pirogov Russian National Research Medical University, 117513 Moscow, Russia; 4 G.N. Gabrichevsky Research Institute for Epidemiology and Microbiology, 125212 Moscow, Russia; 5 Department of Neurology, Neurosurgery and Medical Genetics, N.I. Pirogov Russian National Research Medical University, 117513 Moscow, Russia

**Keywords:** multiple sclerosis, neuroinflammation, curcumin, add-on therapy, gut microbiota, microbiota markers

## Abstract

**Background**: Multiple sclerosis (MS) is a chronic autoimmune disease of the central nervous system and is the leading cause of neurological disability. Currently, the main strategy for MS therapy is the use of disease-modifying therapies (DMTs). If low-efficacy DMTs are ineffective, patients are transferred to high-efficacy DMTs, which possess more severe side effects associated with immunosuppression. Therefore, the search for new add-on therapies for MS that can enhance the effect of low-efficacy DMTs is relevant. Curcumin, being a natural polyphenol, has immunoregulatory properties and a favorable safety profile. In addition, micellar forms of curcumin can increase its bioavailability. We studied the effect of micellar curcumin on clinical and laboratory parameters in patients with MS receiving low-efficacy DMTs (IFN-β). **Methods**: Sixty patients with MS and a suboptimal response to IFN-β were randomized (1:1) into two groups: the IFN-CUR group, which received add-on therapy with micellar curcumin (containing curcumin and Tween 80 as a solubilizer) for 6 months, and a control group (IFN group), which received IFN-β alone. The 6-month treatment period was followed by a subsequent 6-month follow-up off curcumin treatment (DMTs only). **Results**: The proportion of patients without relapses in the curcumin add-on group increased significantly after 6 months (from 57% to 90%, *p* = 0.007), and the risk of exacerbation was significantly lower compared to the control group (HR = 0.2; *p* = 0.03). The treatment was associated with EDSS score stabilization, a positive effect on depression (*p* = 0.05), and a reduction in plasma IFN-γ levels (*p* = 0.02). A decreasing trend in MRI lesion activity and reductions in specific microbiota-related markers, including the *Eggerthella lenta*-associated marker (i16a), were also observed. In ex vivo cultures, curcumin significantly inhibited IL-6 production in macrophages derived from patients with multiple sclerosis (MS) and healthy donors. **Conclusions**: Add-on therapy with micellar curcumin may enhance the efficacy of IFN-β, improving clinical outcomes and modulating inflammatory and microbial parameters in MS patients with a suboptimal response to IFN-β treatment.

## 1. Introduction

Multiple sclerosis (MS) is a chronic autoimmune neurodegenerative demyelinating disorder of the central nervous system. MS is one of the leading neurological causes of disability among young adults [1]. In recent decades, the introduction of disease-modifying therapies (DMTs) has led to substantial improvements in MS treatment. DMTs are classified into several lines with increasing immunosuppressive potency. The most commonly used first-line DMTs include glatiramer acetate and interferon-beta (IFN-β). When first-line DMTs prove ineffective, patients are transitioned to second-line therapies, which, while more potent, are associated with higher costs and a greater risk of adverse effects [2].

Therefore, it is important to increase the efficiency of first-line DMTs to reduce the need for escalation to second-line therapies. This is especially relevant for patients with a suboptimal response to treatment, who will need to receive second-line DMTs in case of further worsening of MS symptoms. In this context, we can highlight the concept of complementary or add-on therapy, which can be used in conjunction with first-line therapy to improve treatment outcomes. Probably the substances used for such therapy should have immunoregulatory and anti-inflammatory activity, as well as a favorable safety and tolerability profile. There are various examples of add-on therapies that are studied in MS, for instance, vitamin D, antidepressants, statins, metformin, and beta-blockers [3,4,5,6,7]. Also, there has been evidence that the use of polyphenolic compounds, particularly curcumin, leads to an increase in the anti-inflammatory effect of DMTs in patients with MS.

Curcumin (diferuloylmethane), a naturally occurring polyphenol found in the rhizome of *Curcuma longa*, exhibits a range of pharmacological properties, including anti-inflammatory and neuroprotective effects. Studies have consistently highlighted curcumin’s favorable safety profile. However, like many polyphenols, curcumin has low bioavailability, resulting in plasma concentrations significantly below those required for its pharmacological effects [8,9,10].

Recent advances have led to the development of strategies to enhance curcumin’s bioavailability. One of the most effective approaches involves the use of micellar (nanoemulsion) formulations of curcumin. A common approach to formulating curcumin nanoemulsions is the use of surfactants. In particular, micellar formulations prepared with the surfactant polysorbate 80 (Tween 80) have been shown to increase plasma curcumin concentrations in healthy individuals to 0.4–3 µM, levels comparable to those demonstrating anti-inflammatory effects in vitro. Importantly, these formulations maintain curcumin’s favorable safety profile [9,11]. In the present study, a micellar curcumin formulation based on Tween 80 was used.

Given these developments, there is growing interest in exploring the effects of curcumin formulations with enhanced bioavailability in chronic inflammatory and autoimmune conditions, including MS. Preliminary results from clinical trials have reported positive outcomes for various curcumin formulations as add-on therapies for MS [12,13,14,15].

As mentioned, curcumin has anti-inflammatory and neuroprotective effects. These effects are largely mediated through the inhibition of key inflammatory signaling pathways, including NF-κB and MAPK, resulting in reduced expression of proinflammatory cytokines such as IL-6, TNF-α, and IL-1β [16,17]. Curcumin has also been shown to modulate the JAK/STAT pathway and inhibit NLRP3 inflammasome activation, thereby potentially attenuating innate immune responses involved in multiple sclerosis pathogenesis [17,18]. In addition, it exhibits antioxidant activity via activation of the Nrf2 pathway, thereby enhancing the expression of cytoprotective enzymes, which is considered to be important for reducing oxidative stress–induced neuronal damage [19]. Curcumin may further promote neuroprotection by supporting mitochondrial function and reducing microglial activation [20]. Moreover, modulation of the gut microbiota may represent an additional mechanism of action of curcumin in MS. Dysbiosis observed in MS is associated with increased autoimmune inflammation [21,22], whereas curcumin may promote beneficial commensals and restore short-chain fatty acid production, thereby contributing to immune homeostasis and reduced neuroinflammation [23].

In light of these data, we hypothesize that curcumin may serve as an add-on therapy for MS, potentially improving disease course and clinical outcomes in patients with a suboptimal response to IFN-β. Based on these considerations, we conducted a study to evaluate the impact of dietary supplementation with micellar curcumin in MS patients with suboptimal responses to IFN-β therapy. We assessed clinical outcomes and, to explore potential mechanisms, evaluated plasma cytokine levels and circulating microbiota markers in patients receiving curcumin. To further investigate possible cellular mechanisms, we evaluated the ex vivo functional activity of macrophages by measuring IL-6 production in cell cultures derived from MS patients and healthy donors.

## 2. Materials and Methods

This prospective randomized comparative study evaluated the effects of curcumin on clinical, immunological, and microbiota parameters in MS patients. The study protocol was approved by the local ethics committee of the Federal Center for Brain and Neurotechnology. All patients gave written informed consent to participate in the study. The study was performed in accordance with good clinical practice and the Declaration of Helsinki [24]. The study is registered in the https://gisnauka.ru/ (accessed on 13 April 2026) database (122022100106-9).

The number of patients in each group was calculated to ensure the possibility of determining the statistical significance of differences at a significance level of α = 0.05 and statistical power of β = 0.8, using two-sided statistical tests. All 60 patients in this study had relapsing-remitting MS (RRMS) and were receiving IFN-β therapy. No difference was found in their demographic baseline characteristics (Table 1). All participants had a suboptimal response to DMTs (relapse in the previous 6 months or activity on MRI (T1 gadolinium-enhancing Gd^+^ lesions or new/enlarging T2 lesions on MRI, combined unique active (CUA) lesions) in the previous 12 months). Participants were randomized into 2 groups of 30 people (patients) each. Randomization of patients was performed using cryptographic algorithms for generating pseudo-random numbers. The first group (IFN-CUR) received curcumin (in addition to IFN-β therapy) in an enhanced bioavailability formulation (micellar curcumin, Evalar, Russia), 80 mg per day for 6 months; the second group (IFN) received DMTs therapy alone (IFN-β therapy). Ingredients per curcumin capsule: polysorbate 80 (Tween 80), capsule shell components, and turmeric extract (providing 40 mg of curcumin). After curcumin intake was discontinued, patients were followed up for another 6 months; the total observation period was 12 months. Detailed clinical characteristics of the patients are presented below (Table 1 and Table 2).

At the first visit (month 0), a medical history was taken, neurological status was assessed with an evaluation of functional systems on the J.F. Kurtzke scale, and the severity of the disease in patients with RS was determined on the extended EDSS disability scale [25]. Radiological activity (T1 Gd^+^ lesions or new/enlarging T2 lesions on MRI, CUA lesions) was assessed using MRI of the brain and spinal cord on at least a 1.5 Tesla system as part of routine clinical monitoring of patients. CUA-lesion counts, defined as the sum of T1 gadolinium-enhancing (Gd^+^) lesions and new or enlarging active T2 lesions (without T1 Gd^+^), were compared between the postbaseline and the baseline period. Neuropsychological testing was carried out to identify factors that might influence patients’ commitment to add-on therapy, including an assessment of depression using the Beck scale [26] and an assessment of cognitive function using the MoCA (Montreal Cognitive Assessment) test. A blood sample was also taken at this visit, followed by plasma collection for further assessment of immunological and microbiota parameters (IL-6, IFN-γ, levels of microbiota markers).

The second visit (month 6) included an assessment of all the indicators assessed at the first visit, as well as an assessment of biochemical blood parameters (liver enzymes, bilirubin), which were routinely performed by the patients as part of compliance with the risk management plan for DMTs. Plasma levels of the cytokines (IL-6 and IFN-γ) and levels of microbiota markers were also assessed. For MS exacerbation, we accepted subjective (reported by the patient) and/or objective (revealed during neurological examination) symptoms in the form of worsening of pre-existing neurological symptoms, or the appearance of new neurological symptoms not observed previously, lasting more than 24 h, provided that there is no fever or other signs of an infectious disease.

**Figure 1:**
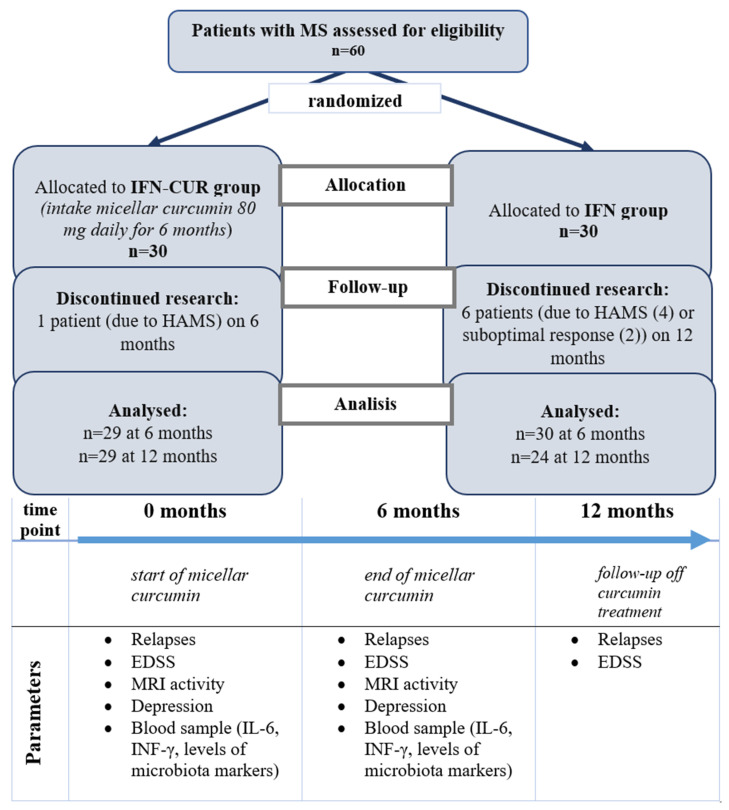
Study of curcumin in people with MS. IFN-CUR is a group of patients taking IFN-β with curcumin add-on therapy. IFN is a group of patients taking IFN-β without curcumin add-on therapy. HAMS—highly active multiple sclerosis.

Patients were clinically followed up for up to 12 months to assess disease progression (exacerbations, EDSS scores). During the follow-up period, exacerbations were recorded and, if necessary, steroid treatment was performed. See Figure 1 for the study design.

### 2.1. Outcomes

The primary endpoint is the time to first documented relapse. Secondary endpoints included proportion of patients without exacerbations, proportion of patients with confirmed disability progression (CDP), T1 Gd^+^ lesions, and combined unique active lesions (CUA lesions) on MRI, and level of depression.

### 2.2. The Analysis of Microbiota Markers

In the current work, we performed the assessment of microbiota markers in the blood of patients who received add-on curcumin with an enhanced bioavailability (IFN-CUR group) and patients who received only treatment with IFN-β (IFN group). We have compared the level of microbiota markers prior to curcumin treatment and after 6 months of curcumin treatment (or 6 months of no curcumin treatment in the control group (IFN group).

Bacteria produce a wide variety of fatty acids with odd numbers of carbon chains, branched chains, and hydroxyl groups (microbiota markers). There are currently more than 250 of them known (in the human body, there are only about 25). Microbiota markers can spread throughout the whole body and can be measured at low levels by gas chromatography with mass spectral detection (GC-MS) [27,28,29]. This method was used in the current study. The high sensitivity of this method allows the detection of microbiota markers in very low concentrations.

The analysis consists of direct extraction of fatty acids, aldehydes, and sterols from the blood, their chromatography separation, and subsequent mass spectrometry detection. Peripheral blood samples were collected from MS patients into EDTA-containing tubes and stored at −80 °C until analysis. The averaged blood samples (40 µL) were dried with the addition of 40 μL of methanol at 80 °C. The analysis was performed as in the article of Osipov and colleagues [30].

Briefly, fatty acids were released as methyl esters as a result of methanolysis. A mixture of the esters in an amount of 2 μL was injected into the Maestro 7820 A with a mass selective detector (5975 series MSD; Agilent Technologies, Santa Clara, CA, USA) GC-MS system. The mass spectrometer was operated in the selected ion monitoring mode. The algorithm for detecting the mass spectral parameters of a biological specimen can detect about 200 known microbial markers, which is sufficient to reveal and assay more than 170 taxons of clinically significant microorganisms at the level of a genus or a species [30].

### 2.3. The Analysis of IL-6 Production by PBMCs and Macrophages

Peripheral blood samples were collected from MS patients and healthy donors into heparin-containing tubes. Peripheral blood mononuclear cells (PBMCs) were isolated from venous blood of patients with RRMS and healthy donors using Ficoll (PanEco, Moscow, Russia) density gradient centrifugation. Patients with RRMS had a suboptimal response to interferon-beta therapy and were not taking curcumin supplements.

Monocytes were isolated from PBMCs by plastic adherence. Briefly, PBMCs were placed in Petri dishes for 1–2 h, after which non-adherent cells were removed. Adherent monocytes were cultured for 6 days in RPMI-1640 medium (PanEco, Moscow, Russia) supplemented with 10% fetal bovine serum (Biosera, Nuaille, France), penicillin/streptomycin (PanEco, Moscow, Russia), and 50 ng/mL granulocyte-macrophage colony-stimulating factor (GM-CSF; Miltenyi Biotec, Bergisch Gladbach, Germany) to induce differentiation into macrophages.

The resulting macrophages or PBMCs were seeded into a 96-well plate. Curcumin (Enzo Biochem, Farmingdale, NY, USA) was dissolved in dimethyl sulfoxide (DMSO; PanEco, Moscow, Russia). Cells were pre-treated with 1 µM curcumin (final DMSO concentration ≤ 0.05%) for 1 h, followed by stimulation with 100 ng/mL lipopolysaccharide (LPS; Sigma-Aldrich, St. Louis, MO, USA; cat. no. 437628). Controls included untreated cells, LPS-only, and vehicle (0.05% DMSO). After 24 h of incubation (37 °C, 5% CO_2_), supernatants were collected and stored at –80 °C. Concentrations of IL-6 were determined using enzyme-linked immunosorbent assay (ELISA) kits (Vector-Best, Moscow, Russia) according to the instructions of the manufacturer. The optical density at 450 nm was measured using a Varioskan LUX multifunctional microplate reader (Thermo Fisher Scientific, Waltham, MA, USA).

### 2.4. Measurement of IL-6 and IFN-γ Levels in Blood Plasma

Plasma levels of IL-6 and IFN-γ in patients with MS and healthy donors were measured using a highly sensitive enzyme-linked immunosorbent assay (ELISA). Peripheral blood samples were collected into EDTA-containing tubes. Following blood collection, samples were centrifuged to obtain plasma, which was then aliquoted and stored at −80 °C until analysis. Cytokine concentrations were quantified using commercial high-sensitivity ELISA kits (Elabscience, Houston, TX, USA) according to the manufacturer’s instructions. The assay detection limits were 0.78 pg/mL for IL-6 and 1.56 pg/mL for IFN-γ. The optical density at 450 nm was measured using a Varioskan LUX multifunctional microplate reader (Thermo Fisher Scientific, Waltham, MA, USA).

### 2.5. Statistical Analysis

The quantitative results were presented as the median and upper and lower quartiles (Q1-Q4), or as the mean ± standard deviation (SD). For statistical analysis and drawing the graphs, GraphPad Prism version 8.0 was used. Paired *t*-test was used to compare the results of some clinical (EDSS, depression) and immunologic studies before and after treatment in the IFN-CUR and IFN group. An unpaired *t*-test was used to compare the statistical differences between the IFN-CUR group and IFN group; between the MS group and the healthy donor group (plasma cytokine levels and level of IL-6 production). To compare the qualitative indicators, the criterion χ2 was calculated. Spearman’s correlation analysis was performed to assess the relationships between variables. The Kaplan–Meier curve was constructed to assess the hazard ratio of the occurrence of an event (clinical exacerbation).

Differences between groups were considered statistically significant at *p* < 0.05.

## 3. Results

### 3.1. Evaluation of Curcumin Effect on Clinical Characteristics of Patients with MS

#### 3.1.1. Relapses

**Figure 2:**
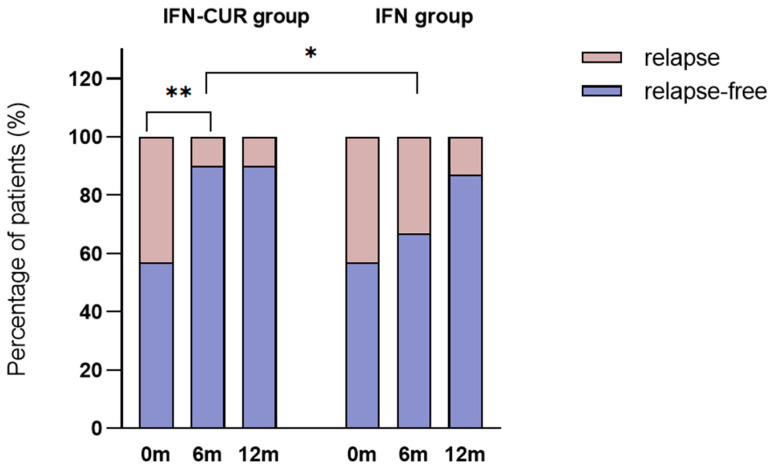
Change in the proportion of patients without exacerbations over 12 months of follow-up. IFN-CUR group—group of patients taking IFN-β with curcumin add-on therapy before enrolment (0 months), after 6 months of curcumin treatment (n = 29), and after an additional 6 months of follow-up (up to 12 months of observation (n = 29). Increase in the proportion of patients without exacerbations at 6 months (from 57% to 90%, *p* = 0.007), unchanged at 12 months—87%. IFN group—group of patients taking IFN-β without curcumin add-on therapy before enrolment (0 months), after 6 (*n* = 30) and 12 months (*n* = 24) of follow-up, respectively. An increase in the proportion of patients without exacerbations from 57% to 67% (*p* = 0.6) at 6 months and to 87% (*p* = 0.1) at 12 months. * p < 0.05, ** *p* < 0.01.

We found the effect of curcumin on the clinical activity of MS. In the IFN-CUR group, the proportion of patients without relapses increased significantly after 6 months (from 57% to 90%, *p* = 0.007). In the IFN group, the proportion of patients without relapses did not increase significantly (57% vs 67%, *p* = 0.6). Up to the 12th month of observation, this indicator tended to increase in both groups without reaching statistical significance (Figure 2).

**Figure 3:**
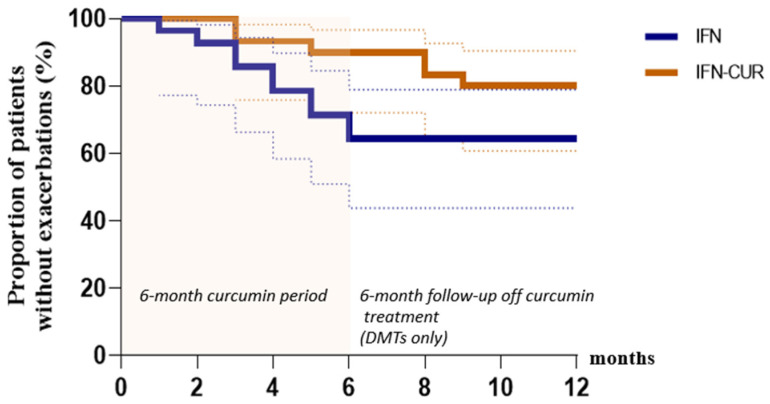
Risk of exacerbation after 12 months of follow-up. IFN-CUR is a group of patients taking IFN-β with add-on curcumin therapy for 6 months (*n* = 30). IFN is a group of patients taking IFN-β without add-on curcumin therapy (*n* = 28). The dashed lines represent confidence intervals.

Also, the Kaplan–Meier survival curve showed a lower risk of exacerbation in the IFN-CUR group. Hazard ratio between treatments after 6 months was 0.3 (95% CI, 0.1–0.9), *p* = 0.03), and after 12 months was 0.5 (95% CI, 0.2–1.3), *p* = 0.1) (Figure 3).

#### 3.1.2. EDSS

We also observed a stabilization of the EDSS score in the curcumin-treated group. Median EDSS scores at 6 months in the IFN-CUR group tended to decrease from 2.0 (1.5; 2.5) at 0 months to 1.5 (1.5; 2.75) at 6 months (*p* = 0.2), while the median EDSS in the comparison group showed no change during the study period. In addition, fewer patients with CDP on the EDSS scale were identified in the IFN-CUR group at 6 months, suggesting the potential of curcumin as a drug capable of influencing progression independent of relapse activity (PIRA) (Table 3).

#### 3.1.3. MRI-Activities

We observed a decrease in MRI activity in the IFN-CUR group at 6 months. There is a trend of decrease in the proportion of patients with T1 Gd^+^ lesions in the IFN-CUR group (from 23% to 14%, *p* = 0.3). It is worth noting that this parameter did not decrease in the control group (Table 3).

We also assessed the proportion of patients with combined unique active lesions (CUA lesions). As is known, CUA lesions are the cumulative number of new T1 Gd^+^ lesions and new/enhancing T2 lesions without double addition. In the IFN-CUR group, the proportion of patients with CUA lesions tended to decrease from 13% to 10% (*p* = 0.7). Conversely, the proportion of patients with CUA lesions in the comparison group exhibited a slight increase at 6 months, rising from 7% to 17% (*p* = 0.2). A comparison between the groups revealed no significant difference (*p* = 0.5) (Table 3).

#### 3.1.4. Depression

The mean level of depression (Beck scale) in the IFN-CUR group decreased from 7.4 ± 4.5 to 6.4 ± 6.0 (*p* = 0.05), whereas in the IFN group, there was no change in depression level.

#### 3.1.5. Safety

We found that curcumin in micellar form was well tolerated during the period of supplementation, with no adverse events, including serious ones that could lead to premature discontinuation of therapy.

### 3.2. Evaluation of Curcumin Effect on Immunological Parameters of Patients with MS

**Figure 4:**
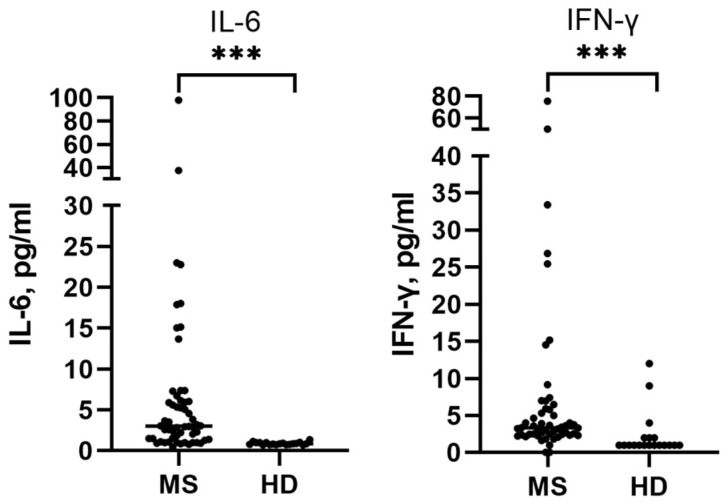
Blood plasma levels of IL-6 and IFN-γ in MS patients and healthy donors. MS—patients with multiple sclerosis, HD—healthy donors. Data are presented as individual values, with the median indicated. *** p < 0.0001.

Using high-sensitive ELISA, we obtained data on baseline levels of proinflammatory cytokines in blood plasma from MS patients and healthy donors. The plasma levels of IFN-γ and IL-6 were significantly higher in MS patients (n = 58)—3.2 (2.3; 5.6) and 3.1 (1.5; 6.3) pg/mL, respectively, compared to healthy donors (n = 19)—1 (1; 2) and 0.85 (0.75; 1.0) pg/mL, respectively (Figure 4, Table 3), which is consistent with literature data.

**Figure 5:**
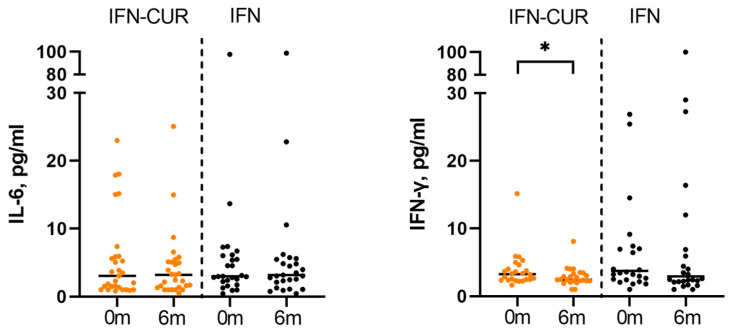
Effect of curcumin on blood plasma levels of IL-6 and IFN-γ in patients with MS. IFN-CUR—group of patients taking IFN-β with add-on curcumin therapy, IFN—comparison group (without taking curcumin). Data are presented as individual values, with the median indicated. * p < 0.05.

We evaluated the effect of curcumin on IL-6 and IFN-γ blood plasma levels of patients with MS. We found that in the IFN-CUR group, the median level of IFN-γ decreased significantly from 2.8 (2.3; 4.4) to 2.4 (2.0; 3.2, *p* = 0.02) pg/mL, whereas in the IFN group, the median level of IFN-γ did not change significantly. No effect of curcumin on IL-6 levels was observed in patients in the IFN-CUR group (3.3 (1.4; 5.9) vs 3.2 (1.5; 5.4), *p* = 0.7) pg/mL, while a slight upward trend in this cytokine was observed in the comparison group (3.0 (2.1; 6.3) vs 3.3 (2.3; 5.6), *p* = 0.5) pg/mL (Figure 5, Table 3).

Detailed results are presented in Table 3.

The correlations between clinical and demographic data and immunological parameters at baseline were also evaluated. Our analysis revealed several slight correlational trends. For instance, IFN-γ level correlated with exacerbations in MS patients in the previous 6 months (r = 0.26, *p* = 0.05).

In addition, we evaluated the effect of initial cytokine production on clinical parameters in MS patients at 6 months. A weak direct correlation was found between the initial IL-6 level (at 0 months) and the appearance of new/enlarging-T2 lesions at 6 months (r = 0.25, *p* = 0.06).

After 6 months of follow-up, we evaluated the correlation between cytokine production (IL-6 and IFN-γ) and clinical parameters in the IFN-CUR and IFN groups. A direct weak correlation was found between IFN-γ level and exacerbations in the IFN-CUR group (*p* = 0.05). Also, in the comparison group, we detected a correlation between the level of depression at 6 months and IL-6 level (r = 0.27, *p* = 0.07).

### 3.3. Curcumin Effect on Microbiota Markers in the Blood of Patients with MS

**Figure 6:**
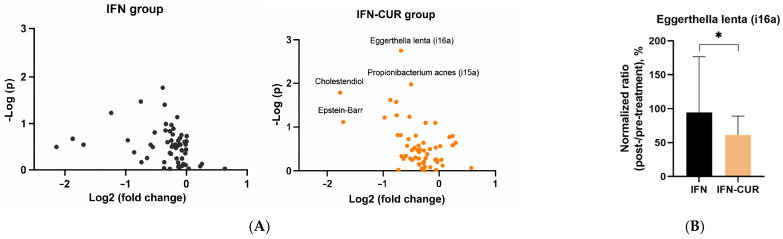
The influence of add-on therapy with micellar curcumin on blood microbiota markers in patients with multiple sclerosis receiving IFN-β. (**A**) Changes in microbiota markers are presented as log2 fold change relative to baseline (6 months vs 0 months). The IFN group includes patients receiving IFN-β alone, whereas the IFN-CUR group includes patients receiving IFN-β with micellar curcumin as add-on therapy. (**B**) Comparison of changes in the *Eggerthella lenta*-associated marker (i16a) between IFN and IFN-CUR groups. Values are expressed as normalized post-/pre-treatment ratios (%), where pre-treatment corresponds to baseline (0 months) and post-treatment to 6 months. Data are presented as median and interquartile range. * p < 0.05.

**Figure 7:**
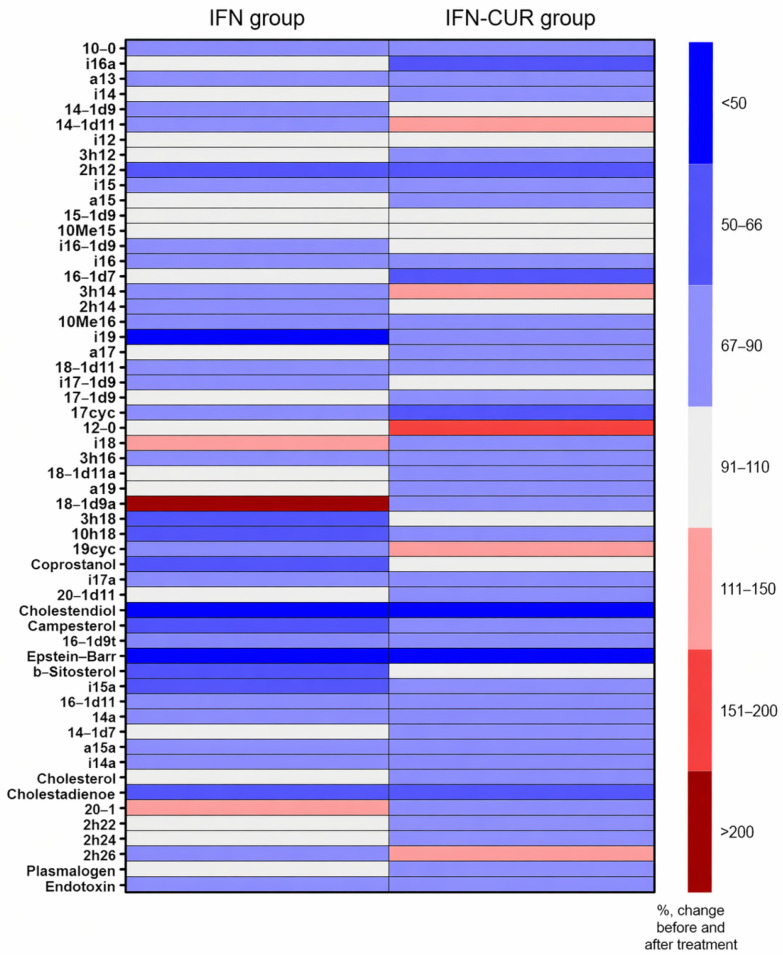
Cluster heat map of changes in microbiota markers in the blood of patients with multiple sclerosis receiving IFN-β (IFN group) or IFN-β and micellar curcumin as add-on therapy (IFN-CUR group). Areas of red or blue indicate that the marker of microbiota is increased or decreased, respectively, compared to the beginning of add-on therapy (6 months compared to 0 months). The white area indicates that there was virtually no change in the microbiota marker level. Substance designations: 17: 1–17—number of carbon atoms, the number after the colon—number of double bonds; h—oxyacid; a,i—at the beginning means branching; cyc—cyclopropanoic acid. For example, ha17: 3-oxyanteisoheptadecanoic acid [32].

We found a decrease in certain microbiota markers in the blood of patients receiving curcumin add-on therapy (Figure 6 and Figure 7).

In the group of patients taking curcumin, markers of viral infections decreased. For instance, we observed a decrease in the content of herpes infection markers. The level of *cholestendiol* (a cholesterol metabolite that was shown to increase in herpes infections [31]) decreased more than threefold. In addition, there is also a tendency for *Epstein–Barr virus* marker (ergostenil-oxi [32]) to decrease in the curcumin group.

Regarding bacterial markers, the *Eggerthella lenta*-associated marker (i16a) decreased significantly in patients receiving curcumin, while no such change was observed in the control group. Importantly, a significant difference between the IFN-CUR and IFN groups was observed for this marker, indicating a reduction only in the curcumin-treated group.

It is worth noting that after a course of curcumin add-on therapy, there was a decrease in the levels of markers, which, according to our previous studies [33], were increased in the CSF of patients with MS in remission, compared to healthy donors (in particular, the *Propionibacterium acnes* marker (i15a) and the marker of *Epstein–Barr virus*).

### 3.4. Effect of Curcumin on Induced Cytokine Production by PBMCs and Macrophages from Patients with MS and Healthy Donors

This study investigated the effect of curcumin on the induced production of IL-6 by PBMCs and macrophages derived from patients with MS and healthy donors.

Curcumin had no significant effect on IL-6 production by PBMCs in either group.

**Figure 8:**
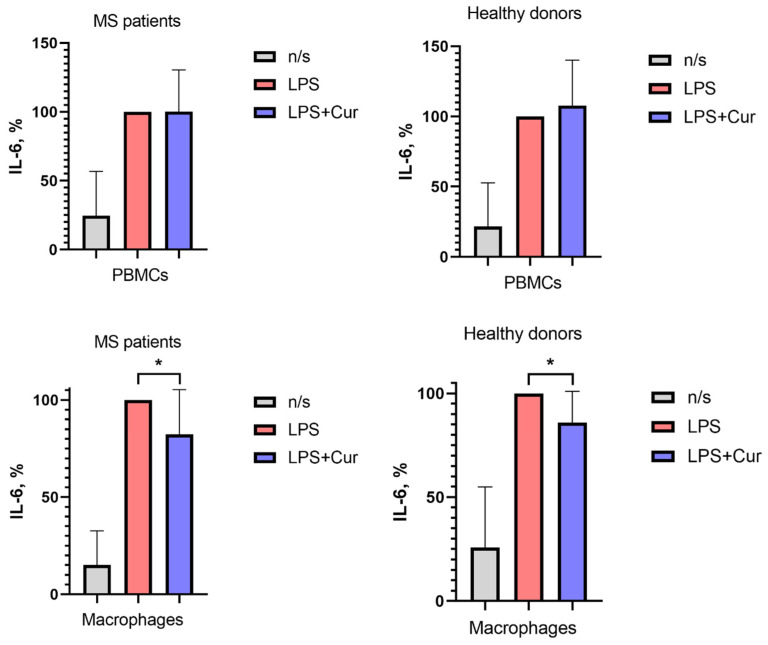
The influence of curcumin on IL-6 production by activated PBMCs and macrophages in MS patients and in healthy donors. Data are presented as means and SD. Macrophages were cultured from monocytes obtained from MS patients in clinical remission and from healthy donors. PBMCs and macrophages were activated with LPS with or without the addition of curcumin at a concentration of 1 μM (LPS+Cur). After 24 h, the supernatants were collected and stored at –80 C until analysis. IL-6 concentrations were then quantified using a commercial ELISA kit. The boxes in the graphs correspond to the means, and the whiskers indicate the SD. * p < 0.05.

In macrophages, curcumin reduced stimulated IL-6 production in both MS patients and healthy donors (Figure 8).

## 4. Discussion

Our research demonstrated that micellar curcumin is well-tolerated by patients with RRMS, with no serious adverse effects leading to treatment discontinuation during add-on therapy. These results are in line with other data showing the safety of curcumin [7,10,11,12,13].

### 4.1. Key Clinical and Radiological Findings

After the 6-month treatment period, curcumin significantly reduced the risk of clinical relapse compared to the control group. A similar, though non-significant, trend was observed in the control group. During the subsequent 6-month follow-up period off treatment, relapse rates demonstrated a declining trend in both groups; however, this trend did not reach statistical significance.

Notably, previous studies have highlighted curcumin’s anti-inflammatory effects on radiological outcomes in MS patients [14,15]. In our study, the proportion of patients with CUA lesions in the IFN-CUR group exhibited a decreasing trend at month 6, whereas an increasing trend was observed in the comparison group. These results are consistent with the findings of Petracca et al., who reported a lower proportion of patients with CUA lesions at month 12 in the IFN–curcumin group compared to the IFN–placebo group; however, this difference was not sustained at month 24, highlighting the importance of long-term follow-up [14].

Regarding disability, patients receiving micellar curcumin maintained stable EDSS scores, whereas the control group exhibited an increase in disability, reflected by a rise in the 75th percentile of EDSS scores, which is consistent with the findings of Dolati et al. [11,12]. In addition, curcumin supplementation was associated with an improvement in depression scores, in line with both clinical and animal model data [34,35].

Taken together, these clinical and radiological outcomes support our hypothesis that micellar curcumin may serve as an effective add-on therapy in MS, potentially improving disease course and clinical outcomes in patients with a suboptimal response to IFN-β.

### 4.2. Effects on Cytokines and Microbiota Markers

Beyond clinical outcomes, we assessed curcumin’s effects on plasma cytokine levels and the level of microbiota markers. Previous studies [11,12,13] suggest that curcumin reduces cytokine production. Similarly, we noted its inhibitory effect on IFN-γ plasma levels in MS patients. While IL-6 levels remained unchanged with curcumin, the untreated group showed a trend toward increased IL-6. It is also important to note that, in our study, plasma levels of IFN-γ and IL-6 were significantly higher in MS patients with suboptimal response to therapy compared to healthy donors. This finding is consistent with the presence of chronic inflammation in MS, which may represent one of the key mechanisms underlying its pathogenesis.

Among the observed changes in microbiota-related markers in patients receiving curcumin, reductions were observed in the *Eggerthella lenta*-associated marker, as well as in herpes virus–associated markers, including the Epstein–Barr virus–associated marker. Usually, microbiota data are characterized by high variability, which means that the data we obtained can only serve as a basis for hypotheses that need to be verified.

### 4.3. Hypothesis on the Mechanism of Action

Recently, increasing evidence has emerged to support the hypothesis that MS is caused by an immune response to Epstein–Barr virus antigens. It has been shown that in normal conditions, autoreactive cells that are cross-specific to viral antigens and myelin sheath antigens can be removed by effector cells (especially natural killer cells). In MS, impairments in the elimination of autoreactive cells have been found, as extensively described in the work of Vietzen and coauthors [36]. According to our data, the potential therapeutic efficacy of curcumin in MS may be attributed, in part, to its ability to reduce the Epstein–Barr virus load within the host organism. Consistent with this, studies have shown that curcumin downregulates the expression of Epstein–Barr nuclear antigen 1 (EBNA1), which is essential for viral latency maintenance [37].

A decrease in the *Eggerthella lenta*-associated marker in patients receiving curcumin may also be clinically relevant. *Eggerthella lenta* has been associated with various autoimmune diseases, including MS [22,38,39]. These Gram-positive anaerobic bacteria have been reported to increase the abundance of Th17 cells, which play a key role in many autoimmune diseases. A recent study by Yern-Hyerk Shin and colleagues described the mechanism by which *Eggerthella lenta* may enhance inflammatory responses in a cell- and antigen-independent fashion. Similar to a natural signaling system, this mechanism activates RORγt through a plasmalogen-mediated signal involving DAMP (damage-associated molecular patterns)-sensing receptors [40]. Therefore, a reduction in the *Eggerthella lenta*-associated marker observed in the curcumin group may contribute to the attenuation of proinflammatory signaling, potentially supporting its beneficial effects.

There are numerous mechanisms underlying the anti-inflammatory action of curcumin. One of them is its ability to suppress the production of proinflammatory factors. However, many in vitro studies use curcumin concentrations that are not achievable in humans. According to clinical studies, oral intake of micellar curcumin can increase its plasma concentration up to approximately 0.4–3 µM. In the present study, we have demonstrated that curcumin at a physiologically relevant concentration (1 µM) suppresses IL-6 production by macrophages.

Additionally, a potential synergism between curcumin and IFN-β [41] may contribute to the observed clinical effects in MS patients. The effects of IFN-beta in MS are mediated by the regulation of various interferon-stimulated genes, resulting in broad immunomodulatory activity. These include the down-regulation of Th1 cytokines (e.g., TNFα, IFN), MHC-II expression on antigen-presenting cells, and the up-regulation of apoptotic markers on B lymphocytes, which promotes the death of memory B cells [42]. According to various studies, curcumin exerts its anti-inflammatory effects primarily by modulating signaling pathways, such as inhibiting nuclear factor-κB (NF-κB) activation and regulating the mitogen-activated protein kinase/extracellular signal-regulated kinase (ERK) and Janus kinase/signal transducer and activator of transcription (JAK/STAT) pathways [17]. As these pathways are linked to proinflammatory cytokine signaling, their modulation may synergize with the IFN-beta-mediated reduction in Th1 cytokine expression. This potential overlap in mechanisms may contribute to the enhanced clinical outcomes observed in the curcumin add-on group.

## 5. Conclusions

The preliminary results of this pilot study suggest that micellar curcumin is a safe add-on therapy that can enhance the effectiveness of interferon-beta (IFN-β) in patients with MS.

However, it is important to note that this study had a number of limitations. First, the study was not placebo-controlled. However, it should be noted that it can be difficult to create a placebo that completely matches the taste of commercially available curcumin preparations. Therefore, there is a significant risk of unblinding the study. Second, the six-month follow-up period and sample size are probably insufficient to evaluate the efficacy of the add-on therapy regarding several clinical indicators.

Nevertheless, our pilot study on the efficacy of micellar curcumin in patients with suboptimal responses to first-line DMTs suggests that micellar curcumin may delay switching to second-line DMTs. Further studies with larger sample sizes are needed to confirm this hypothesis and determine the long-term effectiveness of curcumin as an add-on therapy for MS.

## Figures and Tables

**Table 1:** Characteristics of groups of people with MS. Data are presented as M ± SD, where M is the mean and SD is the standard deviation, and as absolute and relative values (%).

Parameters		MS Patients		*p*-Value Between Groups
		IFN-CUR Group (*n* = 30)	IFN Group (*n* = 30)	
Gender	male	8 (27%)	16 (53%)	
	female	22 (73%)	14 (47%)	
Age, years (M ± SD)		33.1 ± 7.2	36.0 ± 9.5	*p* = 0.2
Duration of disease, years (M ± s)		6.8 ± 4.9	7.9 ± 4.7	*p* = 0.3
DMTs	Peg-IFN-β 1a 125 µg	10 (33%)	12 (40%)	
	IFN-β 1b 250 µg	10 (33%)	14 (17%)	
	IFN-β 1a 44 µg	5 (17%)	0 (0%)	
	IFN-β 1a 30 µg	5 (17%)	4 (13%)	
EDSS (M ± SD)		2.0 (1.5; 2.5)	1.5 (1.5; 2.5)	*p* = 0.5
Percentage of patients without relapses, %		57%	57%	*p* = 0.9
Percentage of patients with CDP, %		0%	0%	*p* = 0.9
Percentage of patients with T1 Gd^+^ lesions, %		23%	23%	*p* = 0.9
Percentage of patients with CUA lesions, %		13%	7%	*p* = 0.4
Beck scale, (M ± SD)		7.3 ± 4.6	7.6 ± 5.3	*p* = 0.9
IL-6, pg/mL **^#^**		3.3 (1.4; 5.9)	3.0 (2.1; 6.3)	*p* = 0.5
IFN-γ, pg/mL **^#^**		2.8 (2.3; 4.4)	3.6 (2.4; 8.7)	*p* = 0.06

EDSS—extended disability status scale; DMTs—disease-modifying therapy; IFN-β—interferon-beta; IFN-γ—interferon-gamma; IL-6—interleukin-6; IFN-CUR group and IFN group—groups of patients who received or did not receive additional curcumin therapy, respectively; MS patients—patients with multiple sclerosis; M ± SD—mean and standard deviation. ^#^ Data are presented as median (25th; 75th percentile).

**Table 2:** Inclusion and exclusion criteria.

Inclusion Criteria	Exclusion Criteria
1. Availability of a signed Informed Voluntary Consent (IVC) form. 2. Age between 18 and 50 years at the time of enrolment. 3. The patient’s willingness to cooperate and ability to meet the requirements imposed on participants in this study. 4. Confirmed diagnosis of RRMS according to McDonald’s criteria (2017 version). 5. EDSS score of 1 to 5 points inclusive at the time of enrolment. 6. Treatment with IFN-β drugs for at least 12 months. 7. Suboptimal response to the previous one-year course of IFN-β (the presence of relapses in the previous 6 months) or the appearance of T1 Gd^+^ lesions or new/enhancing T2 lesions on MRI.	1. The presence of primary progressive MS (PPMS) and secondary progressive MS (SPMS) at the time of enrolment. 2. Patients receiving highly effective DMTs. 3. Pregnancy, lactation, or intent to become pregnant during the study. 4. Severe physical or mental illness, the presence of which may affect the results of the study. 5. Alcohol or other psychoactive substance abuse. 6. Systemic corticosteroid therapy for 3 months prior to enrolment. 7. Regular vigorous exercise. 8. Presence of cognitive impairment according to the MoCA scale (<26 points). 9. The presence of moderate and severe depression on the Beck scale (> 19 points). 10. The presence of acute inflammatory diseases. 11. Refusal by the patient to continue participation in the study.

EDSS—extended disability status scale; DMTs—disease-modifying therapy; IFN-β—interferon-beta; MRI—magnetic resonance imaging; MoCA—Montreal Cognitive Assessment; RRMS—relapsing-remitting multiple sclerosis.

**Table 3:** Clinical and immunological characteristics of MS patients before and after participation in the study.

Parameters	IFN-CUR Group			IFN Group		
	0 Month (*n* = 30)	6 Month (*n* = 29)	12 Month (*n* = 29)	0 Month (*n* = 30)	6 Month (*n* = 30)	12 Month (*n* = 24)
Percentage of patients without relapses, %	57%	90%	87%	57%	67%	87%
		*p* = 0.007	*p* = 0.007		*p* = 0.6	*p* = 0.6
EDSS ^#^	2.0 (1.5; 2.5)	1.5 (1.5; 2.75)	1.5 (1.0; 2.75)	1.5 (1.5; 2.5)	1.5 (1.5; 2.625)	1.5 (1.5; 2.875)
		*p* = 0.2	*p* = 0.2		*p* = 0.03	*p* = 0.03
Percentage of patients with CDP, %	0	3%	3%	0	10%	10%
			*p* = 0.2			*p* = 0.4
Percentage of patients with T1 Gd^+^ lesions, %	23%	14%		23%	23%	
		*p* = 0.3			*p* = 0.9	
Percentage of patients with CUA lesions, %	13%	10%		7%	17%	
		*p* = 0.7			*p* = 0.2	
Depression, M ± SD	7.4 ± 4.5	6.4 ± 6.0		7.7 ± 5.3	7.9 ± 6.0	
		*p* = 0.05			*p* = 0.7	
IL-6, pg/mL ^#^	3.3 (1.4; 5.9)	3.2 (1.5; 5.4)		3.0 (2.1; 6.3)	3.3 (2.3; 5.6)	
		*p* = 0.7			*p* = 0.5	
IFN-γ, pg/mL ^#^	2.8 (2.3; 4.4)	2.4 (2.0; 3.2)		3.6 (2.4;8.7)	2.9 (2.1; 10.7)	
		*p* = 0.02			*p* = 0.1	

IFN-CUR—group of patients taking IFN-β with add-on curcumin therapy, IFN—comparison group (without taking curcumin). CDP—Confirmed Disability Progression. ^#^ Data are presented as median (25th; 75th percentile). *p*-value was evaluated between months 0 and 6, as well as between months 0 and 12.

## Data Availability

Data available on request due to restrictions.

## Abbreviations

The following abbreviations are used in this manuscript:

CDP	Confirmed disability progression
CUA lesions	Combined unique active lesions
DMTs	Disease-modifying therapy
EDSS	Extended disability status scale
GC-MS	Gas chromatography with mass spectral detection
HAMS	Highly active multiple sclerosis
IFN-β	Interferon-beta
IFN-γ	Interferon-gamma
IFN-CUR	Group of patients taking IFN-β with add-on curcumin therapy
IL	Interleukin
IVC	Informed Voluntary Consent
JAK/STAT	Janus kinases/signal transducer and activator of transcription proteins signaling pathway
MAPK	Mitogen-activated protein kinase
MoCA	Montreal Cognitive Assessment
MS	Multiple sclerosis
MRI	Magnetic resonance imaging
NF-κB	Nuclear factor kappa B
Nrf2	Nuclear factor erythroid 2-related factor 2
PBMCs	Peripheral blood mononuclear cells
Peg-IFN-β 1a	Peginterferon-β 1a
PIRA	Progression independent of relapse activity
RRMS	Relapsing-remitting multiple sclerosis
SPMS	Secondary progressive multiple sclerosis
TNF-α	Tumor necrosis factor α
